# 血管性血友病诊断与治疗中国指南（2022年版）

**DOI:** 10.3760/cma.j.issn.0253-2727.2022.01.001

**Published:** 2022-01

**Authors:** 中华医学会血液学分会血栓与止血学组

一、概述

血管性血友病（von Willebrand disease, VWD）是最常见的遗传性出血性疾病。血管性血友病因子（von Willebrand Factor, VWF）基因突变引起血浆VWF数量减少或质量异常是VWD的主要致病机制[1]。

VWF由血管内皮细胞与巨核细胞合成。VWF的主要作用：①与血小板膜糖蛋白Ⅰb（GPⅠb）及内皮下胶原结合，介导血小板黏附至血管损伤部位；②作为凝血因子Ⅷ（FⅧ）的载体，具有稳定FⅧ的作用。根据VWD发病机制和临床表现，VWD主要分为三种类型（表1）[1]–[2]。

**表1:** 血管性血友病（VWD）的发病机制和分型

分型	特征
1型	VWF量减少
1C	1型VWD中一个特殊亚型，VWF清除增快导致VWF半衰期显著缩短
2型	VWF质的缺陷
2A	缺乏VWF高分子多聚物，与血小板黏附活性降低
2B	VWF与GPⅠb亲和力增加，导致VWF高分子多聚物减少
2M	VWF与血小板黏附活性降低，但VWF多聚体分布正常
2N	VWF与FⅧ亲和力降低，导致FⅧ活性显著下降
3型	VWF量显著减少或缺如

注：VWF：血管性血友病因子；GPⅠb：血小板膜糖蛋白Ⅰb；FⅧ：凝血因子Ⅷ

二、VWD的流行病学及遗传特点

如果按照血浆VWF水平降低来诊断VWD，预计发病率为1％[3]。但从有明显出血症状同时伴VWF水平降低判断，VWD的发病率为1/1000[2]–[3]。VWD患者的遗传方式存在很大的异质性，不同分型VWD的遗传规律各有不同（表2）。

**表2:** 血管性血友病（VWD）的分型与临床表现

临床表现	1型	2A型	2B型	2M型	2N型	3型
遗传方式	常染色体显性或不全显性	常染色体显性或常染色体隐性	常染色体显性	常染色体显性或常染色体隐性	多为常染色体隐性	常染色体隐性
出血倾向	轻、中度	多为中度，个体差异大	多为中度，个体差异大	多为中度，个体差异大	多为中度，个体差异大	重度
病理特征	VWF数量部分减少	与血小板黏附降低	与血小板GPⅠb亲和力增加	VWF多聚体正常，与血小板黏附降低	与FⅧ亲和力明显降低	VWF完全缺乏
VWF∶Ag	减低	减低或正常	减低或正常	减低或正常	多正常	缺如[4]–[5]（<3%）
VWF∶RCo	减低	减低	减低	减低	多正常	缺如（<3%）
FⅧ∶C	减低	减低或正常	减低或正常	减低或正常	显著减低	显著减低
VWF∶RCo/VWF∶Ag比值	>0.7	<0.7[4]	<0.7[4]	<0.7[4]	>0.7	-
RIPA	减低	减低	增加	减低	多正常	缺如
VWF多聚体	正常	异常（缺乏大、中分子多聚物）	异常（缺乏大分子多聚物）	正常	正常	无
DDAVP试验	有效，多聚体增多	部分有效，中分子多聚体增多	致血小板减少	部分有效，多聚体增多	部分有效，多聚体增多	无效

注：VWF：血管性血友病因子；GPⅠb：血小板膜糖蛋白Ⅰb；FⅧ：凝血因子Ⅷ；VWF∶Ag：VWF抗原；VWF∶RCo：瑞斯托霉素辅因子活性；FⅧ∶C：FⅧ促凝活性；RIPA：瑞斯托霉素诱导的血小板聚集；DDAVP：去氨加压素

三、临床表现特征

1. 自幼发病，以皮肤、黏膜出血为主，表现为皮肤瘀点瘀斑、鼻出血和齿龈出血，女性月经增多。严重者可发生内脏出血。关节、肌肉血肿少见。

2. 多为自发性出血或外伤、围手术期出血过多。

3. 在出血程度上有较大的个体差异，部分1型VWD患者无自发性出血表现。

4. 部分患者有出血家族史，有家族史者符合常染色体显性（或不全显性）或隐性遗传规律。

VWF水平受种族、血型、年龄、炎症、妊娠等多种因素影响。对于出血患者，推荐使用国际血栓与止血学会出血评分工具（ISTH-BAT）进行评估（表3）[6]–[7]，根据评估结果决定是否需要行进一步的实验室检查。

**表3:** 血管性血友病（VWD）出血积分（ISTH-BAT）

出血症状	0分	1分	2分	3分	4分
鼻出血	无或轻微	每年>5次或每次>10 min	仅需要就诊	压迫、烧灼或抗纤溶治疗	输血或替代治疗（血制品或重组FⅦa）或DDAVP
皮肤出血	无或轻微	>5个瘀斑或暴露部位瘀斑>1 cm	仅需要就诊	广泛性出血	自发性血肿需要输血治疗
轻微外伤出血	无或轻微	每年>5次或每次>10 min	仅需要就诊	外科止血	输血或替代治疗或DDAVP
口腔出血	无或轻微	当下就有出血	仅需要就诊	外科止血或抗纤溶治疗	输血或替代治疗或DDAVP
胃肠道出血	无或轻微	当下就有出血（排除消化性溃疡、门脉高压、痔疮和血管发育不良导致的出血）	仅需要就诊	外科止血或抗纤溶治疗	输血或替代治疗或DDAVP
血尿	无或轻微	当下可见的肉眼血尿	仅需要就诊	外科止血，补铁治疗	输血或替代治疗或DDAVP
拔牙出血	无或轻微	出血次数≤拔牙次数的25%，不需要特殊止血	出血次数>拔牙次数的25%，不需要特殊止血	再次缝合或压迫止血	输血或替代治疗或DDAVP
手术后出血	无或轻微	出血次数≤手术次数的25%，不需要特殊止血	出血次数>手术次数的25%，不需要特殊止血	外科止血或抗纤溶治疗	输血或替代治疗或DDAVP
月经增多	无或轻微	仅就诊或频繁更换卫生巾（间隔<2 h）或有血块、月经量大或月经失血图评分法（PBAC）积分>100分	每年超过2次，影响正常工作或学习或者需要抗纤溶或激素或铁剂治疗	需要激素和抗纤溶药物联合治疗或初潮后至今大于12个月	急性月经增多需要住院或急诊治疗或需要输血或替代治疗或DDAVP或者需要子宫扩张清宫术或子宫内膜消融或子宫切除术
产后出血	无或轻微	仅就诊或使用催产素或恶露>6周	铁剂或抗纤溶治疗	需要输血或替代治疗或DDAVP或需要麻醉下检查或U型气囊填塞子宫	任何需要手术干预的治疗（如子宫切除术、髂内动脉或子宫动脉栓塞术、子宫缝合止血术）
肌肉血肿	无	创伤后不需要治疗	自发性出血，不需要治疗	自发性出血或创伤后需要DDAVP或替代治疗	自发性或创伤后需要手术干预或输血
关节出血	无	创伤后不需要治疗	自发性出血，不需要治疗	自发性出血或创伤后需要DDAVP或替代治疗	自发性或创伤后需要手术干预或输血
中枢神经系统出血	无	-	-	硬膜下出血，需要治疗	颅内出血，需要治疗
其他出血症状	无或轻微	当下就有出血	仅需要就诊	外科止血或抗纤溶治疗	输血或替代治疗或DDAVP

注：ISTH-BAT：国际血栓与止血学会出血评分工具；ISTH-BAT积分男性>3分、女性>5分为异常出血；DDAVP：去氨加压素；FⅦa：凝血因子Ⅶa

四、实验室检查

（一）出血筛选试验

包括全血细胞计数、活化部分凝血活酶时间（APTT）、凝血酶原时间（PT）及血浆纤维蛋白原测定。VWD患者筛选检查结果多正常或仅有APTT延长且可被正常血浆纠正。

（二）VWD诊断试验

1. 血浆VWF抗原（VWF∶Ag）测定。

2. 血浆VWF血小板结合活性测定：①VWF与野生型GPⅠb结合测定（VWF∶GPⅠbR）；②VWF与GPⅠb突变体结合测定（VWF∶GPⅠbM）；③VWF瑞斯托霉素辅因子活性（VWF∶RCo）测定。VWF∶GPⅠbR和VWF∶GPⅠbM检测方法重复性好，受影响因素小，检测下限更低，但是国内各医院尚未常规开展。VWF∶RCo与前两项试验相比虽然变异系数偏高，但仍保持了较好的一致性，且国内医院已开展检测。

3. FⅧ促凝活性（FⅧ∶C）测定。

（三）VWD分型诊断试验

1. 血浆VWF多聚体分析。

2. 瑞斯托霉素诱导的血小板聚集（RIPA）。

3. 血浆VWF胶原结合试验（VWF∶CB）。

4. 血浆VWF与FⅧ结合活性（VWF∶FⅧB）。

5. 去氨加压素（1-deamino-8-D-arginine vasopressin, DDAVP）试验：推荐对新诊断的1型和2型VWD患者（2B型除外）进行DDAVP试验。静脉注射DDAVP（剂量参见“预防与治疗”部分），注射后1、4 h后检测FⅧ∶C和VWF水平（包括VWF∶Ag和VWF活性），如DDAVP注射后1 h两者增高大于基础值2倍，且FⅧ∶C和VWF水平均上升至>30％～<50％，为DDAVP试验部分有效；FⅧ∶C和VWF水平均上升至≥50％，则判断为完全有效[4],[8]。如患者注射DDAVP后1 h VWF水平显著升高，但注射后4 h VWF水平快速下降，下降幅度>30％提示VWF清除增快，有助于1C型VWD诊断[4]。以上试验详细结果见表2。

6. VWF前导肽（VWF propeptide, VWFpp）测定。

7. VWF基因测序：随着二代基因测序技术的发展，VWF基因测序在VWD的诊断中发挥了重要作用，2型和3型VWD患者中VWF基因突变检出率高达90％以上。此外，对于2N型VWD和血友病A、2B型VWD和血小板型VWD的鉴别诊断，VWF基因测序尤为重要[9]。但是对于1型VWD，仅有62％的患者可检测到VWF基因突变[2]。

ISTH-BAT积分异常者、有显著的出血病史或凝血检查结果异常者以及VWD患者的直系亲属，建议行VWD诊断和分型诊断试验[4]。

五、诊断

1. 有家族史者符合常染色体显性（或不全显性）或隐性遗传规律。

2. 有自发性出血或外伤、围手术期出血增多史，并符合VWD临床表现特征。

3. 血浆VWF水平<30％，无论有无出血症状，均可诊断。有异常出血表现，VWF水平<50％也可诊断[4]。FⅧ∶C<30％多见于2N型和3型VWD。

4. 排除血友病A、获得性血友病A、获得性von Willebrand综合征（acquired von Willebrand disease, AVWS）、血小板型VWD、遗传性血小板病等。

VWD分型诊断参见表1，诊断流程图见图1。曾明确诊断为1型VWD的患者，即便VWF水平恢复正常，因不能确定患者的出血事件是否随VWF水平的增高而减少，仍应诊断为VWD。

**图1:**
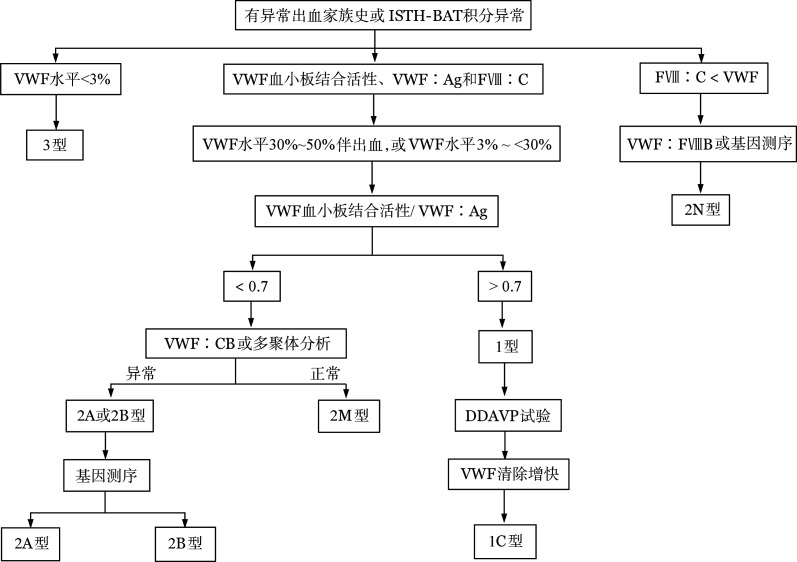
血管性血友病（VWD）诊断流程图 ISTH-BAT：国际血栓与止血学会出血评分工具；VWF：血管性血友病因子；VWF∶Ag：血管性血友病因子抗原；FⅧ：凝血因子Ⅷ；FⅧ∶C：凝血因子Ⅷ促凝活性；VWF∶FⅧB：血浆VWF与FⅧ结合活性；VWF∶CB：血浆VWF胶原结合试验；DDAVP：去氨加压素

六、预防与治疗

在VWD患者出血发作或围手术期，建议应用提升血浆VWF水平的药物或因子行替代治疗，并辅以其他止血药物。同时应根据VWD类型和出血特征选择合适的治疗方法。

（一）DDAVP

DDAVP通过刺激血管内皮细胞释放储备的VWF等机制提升血浆VWF水平。适用于轻中度出血患者，对于1型VWD和部分2型VWD（2A、2M、2N型）有效，对3型VWD无效，2B型VWD慎用。推荐DDAVP试验有效者使用。推荐剂量0.3 µg/kg，最大剂量不超过20 µg。用30～50 ml生理盐水稀释后缓慢静脉注射（至少30 min）。间隔12～24 h可重复使用，但多次使用后疗效下降。该药也可皮下或鼻腔给药（DDAVP鼻喷剂）。DDAVP的不良反应有面部潮红、头痛、心率加快等，反复使用可发生水潴留和低钠血症，需限制液体摄入；小于2岁的婴幼儿、妊娠妇女、癫痫及有活动性心脑血管疾病的老年患者禁用。

（二）替代治疗

适用于中重度出血或围手术期的各型VWD患者以及DDAVP治疗无效患者。选用血源性含VWF的FⅧ浓缩制剂或血源性/重组VWF制剂；如条件限制也可使用冷沉淀或新鲜血浆，但存在输血相关疾病传播风险。剂量依据VWD类型和出血发作特征确定。剂量标定以制剂的VWF∶RCo为准。每公斤体重1 U的VWF∶RCo可使血浆VWF∶RCo提高2 U/dl。推荐剂量详见表4。因重组VWF制剂不含FⅧ，故重症出血患者在首次用药时还需要补充FⅧ。

**表4:** 血管性血友病（VWD）患者替代治疗的推荐方案

手术或出血类型	首次剂量（U/kg）	维持剂量（U/kg）	用法	维持时长（d）
严重出血或大手术	40～60	20～40	每12～24 h 1次	3~14
中度出血或小手术	30～60	20～40	每12～24 h 1次	1~5
轻度出血	20～30	/	/	/

注：/：不适用。轻度出血可单次用药，后续如有持续出血表现，可酌情追加用药

（三）其他治疗

1. 抗纤溶药物：①氨甲环酸：25 mg/kg每日3次口服或15 mg/kg每8 h 1次静脉滴注。②6-氨基己酸：首剂4～5 g静脉滴注，此后1 g/h静脉滴注至出血控制，24 h总量不超过24 g。抗纤溶药物偶有血栓形成危险，血尿者禁用。齿龈出血时可局部使用，也可作为VWD患者出血或手术时的辅助治疗。

2. 局部使用凝血酶或纤维蛋白凝胶对皮肤、黏膜出血治疗有辅助作用。

（四）女性VWD患者的治疗

1. 伴月经增多的VWD患者：首先需排除其他与月经增多相关的妇科疾病。对于没有生育要求的患者，采用性激素治疗（复合激素避孕药或左炔诺孕酮释放宫内缓释系统）或者氨甲环酸。有生育需求的患者以氨甲环酸为首选。如单药治疗效果欠佳，可联合使用DDAVP、替代治疗等[10]。子宫内膜切除术或子宫切除术仅适用于常规治疗无效的VWD患者[11]。反复月经过多患者注意评估缺铁和贫血状态，予以铁剂治疗。

2. 出血性卵巢囊肿：部分女性VWD患者发生出血性卵巢囊肿或黄体破裂出血，引起急腹症。治疗方法包括VWF替代治疗、抗纤溶药物等，重症患者需急症手术治疗。术后口服避孕药可预防复发。

3. 妊娠及分娩：VWD妇女可正常妊娠，但出血、流产发生率增高，多发生于妊娠期前3个月[1]。分娩时可采用神经阻滞麻醉，目标VWF水平为50％～150％，并维持至麻醉结束后6 h。1型VWD（某些2型和3型也适用）产妇，分娩后VWF水平和FⅧ∶C快速下降，存在产后出血风险，需密切观察，必要时口服氨甲环酸以预防出血，用药不影响哺乳[12]。VWD产妇还需评估血栓风险，高风险患者应给予预防治疗（如弹力袜、低分子肝素）。

（五）VWD患者其他特殊情况的治疗

1. 围手术期治疗：VWD患者进行大手术时，患者（如2型和3型）术后仅FⅧ∶C达标仍然不能充分止血，需维持FⅧ∶C和VWF水平保持在50％至少至术后3 d。小手术或有创操作时，推荐用DDAVP或因子浓缩物提升VWF水平大于50％，同时联合氨甲环酸治疗[13]，疗效优于仅提升VWF水平大于50％[14]。大手术是指需要进入胸腹盆腔、可能出现严重出血、涉及关节的手术，还包括拔除第三磨牙以及危及患者生命的手术（颅脑、喉部手术等）。而简单拔牙、门诊手术和大手术之外的手术，则定义为小手术。采用重组VWF制剂行替代治疗时，在手术或操作前需要补充FⅧ。

2. VWD患者的抗血小板或抗凝治疗：当VWD患者合并心血管疾病或血栓性疾病并需要抗血小板或抗凝治疗时，可以行抗血小板或抗凝治疗。但如果抗血小板或抗凝治疗时，VWD患者表现出严重出血倾向，建议加用预防治疗[15]。

（六）VWD的预防治疗

对于频繁出血或者有严重出血病史的VWD患者，推荐预防治疗[16]。预防治疗建议每周至少1次及以上的VWF替代治疗，每次40～80 U/kg，持续至少6个月[14]。

综上所述，VWD患者的出血症状轻重不一，VWD的治疗也应根据患者的具体情况量体裁衣。VWD患者面临手术、妊娠、分娩或心脑血管等疾病时，建议患者至有经验的综合管理中心，由血液专科、手术科室、妇产科、麻醉科或其他相关科室的医师共同权衡利弊，制定合适的治疗方案。
